# Anti-vascular effects of vinflunine in the MAC 15A transplantable adenocarcinoma model

**DOI:** 10.1054/bjoc.2000.1587

**Published:** 2001-01

**Authors:** S E Holwell, B T Hill, M C Bibby

**Affiliations:** Clinical Oncology Unit, University of Bradford, West Yorkshire, BD7 1DP, UK; Division de Cancerologie, Centre de Recherche Pierre Fabre, 17 avenue Jean Moulin, Castres Cedex, 81106, France

**Keywords:** anti-vascular effects, vinflunine, vinorelbine, MAC 15A murine tumour

## Abstract

Anti-vascular effects of the novel *Vinca* alkaloid, vinflunine have been investigated in the MAC 15A transplantable murine colon adenocarcinoma model and compared with those induced by the most recently identified clinically useful third generation *Vinca*. Administration of the maximum tolerated dose of either vinflunine (50 mg kg^−1^) or vinorelbine (8 mg kg^−1^) resulted in significant tumour growth delay with subsequent histological analysis revealing substantial haemorrhagic necrosis. This suggested possible anti-vascular effects and these were confirmed by Hoechst 33342 perfusion studies. Vinflunine, currently undergoing Phase I trials in Europe, was found to be at least as effective as the clinically active vincristine and vinorelbine in this model and, remarkably, produced anti-vascular effects at doses much lower than the maximum tolerated dose. Although vinflunine caused apoptosis in HUVEC monolayer cultures this event did not occur within the first 8 hours of exposure whereas vascular shutdown in vivo was observed within the first 4 hours. © 2001 Cancer Research Campaign http://www.bjcancer.com


British Journal of Cancer (2001) 84(2), 290–295
© 2001 Cancer Research Campaign
doi: 10.1054/ bjoc.2000.1587, available online at http://www.idealibrary.com on IDEAL®

http://www.bjcancer.com

### Anti-vascular effects of vinflunine in the MAC 15A transplantable adenocarcinoma model

**SE Holwell[1], BT Hill[2] and MC Bibby[1]**

[1]Clinical Oncology Unit, University of Bradford, West Yorkshire BD7 1DP, UK; [2]Division de Cancerologie, Centre de Recherche Pierre Fabre, 17 avenue Jean Moulin, 81106 Castres Cedex, France

**Summary** Anti-vascular effects of the novel *Vinca* alkaloid, vinflunine have been investigated in the MAC 15A transplantable murine colon adenocarcinoma model and compared with those induced by the most recently identified clinically useful third generation *Vinca*. Administration of the maximum tolerated dose of either vinflunine (50 mg $kg^{-1}$) or vinorelbine (8 mg $kg^{-1}$) resulted in significant tumour growth delay with subsequent histological analysis revealing substantial haemorrhagic necrosis. This suggested possible anti-vascular effects and these were confirmed by Hoechst 33342 perfusion studies. Vinflunine, currently undergoing Phase I trials in Europe, was found to be at least as effective as the clinically active vincristine and vinorelbine in this model and, remarkably, produced anti-vascular effects at doses much lower than the maximum tolerated dose. Although vinflunine caused apoptosis in HUVEC monolayer cultures this event did not occur within the first 8 hours of exposure whereas vascular shutdown in vivo was observed within the first 4 hours. © 2001 Cancer Research Campaign http://www.bjcancer.com

**Keywords:** anti-vascular effects; vinflunine; vinorelbine; MAC 15A murine tumour

The current status of studies aimed at identifying and developing therapies which specifically compromise the function of the existing neovasculature in solid tumours has been reviewed recently (Chaplin and Dougherty, 1999; Hayes et al, 1999). Although evidence for the therapeutic potential of vascular targeting approaches was first provided over 150 years ago, these authors have highlighted the fact that over the last decade significant research effort has been afforded to the development of therapies that specifically target and damage tumour neovasculature. In considering drug-based approaches to vascular targeting, it is the tubulin-binding agents that have emerged as major players. In early studies with colchicine (Ludford, 1948) and podophyllotoxin (Algire et al, 1955) and, more recently, with vinblastine and vincristine (Baguley et al, 1991; Hill et al, 1993) definite anti-vascular effects were recorded, but these were only achieved at doses approaching the maximum tolerated dose (MTD). The introduction of vinorelbine into the clinic in the mid 1980s and the rapid recognition of its major clinical activities, as reviewed recently (Johnson et al, 1996; Budman, 1997), has led to a resurgence of interest in the *Vinca* alkaloid family of anticancer agents. Indeed, a newly identified derivative, vinflunine, with substitutions in the little exploited region of the catharanthine moiety obtained using superacid chemistry and involving the selective introduction of two fluorine atoms at the 20′ position and the reduction of the 3′,4′ double bond (Fahy et al, 1997), is now undergoing Phase I clinical trials in Europe.

This study was initiated to establish whether vinflunine, documented in terms of both survival prolongation and tumour growth inhibition, as having definite superiority over vinorelbine in a series of experimental animal tumour models (Kruczynski et al, 1998a; Hill et al, 1999), produced any effects on tumour vasculature using the MAC 15A transplantable colon adenocarcinoma. This model has previously been validated for evaluating vascular therapies in preclinical studies (Cowen et al, 1995). Vincristine is included in the study as a control reference compound. Observations were also made on the in vitro effects of vinflunine on human umbilical vein endothelial cells (HUVECs).

*Received 6 March 2000*
*Revised 29 September 2000*
*Accepted 18 October 2000*

*Correspondence to: MC Bibby*

### MATERIALS AND METHODS

### Drugs

Vinflunine, vinorelbine and vincristine were provided by Pierre Fabre Médicament (Castres, France) and were dissolved at the appropriate concentrations using sterile physiological saline and injected intraperitoneally at a volume of 0.1 ml per 10 g body weight.

### Animals

Pure strain male NMRI mice aged 6–8 weeks (B & K Universal, Hull, UK) received CRM diet (SDS, Witham, Essex, UK) and water ad libitum. Mice were kept in cages in an air-conditioned room with regular alternating cycles of light and darkness. All animal procedures were carried out under a project licence issued by the Home Office, London, UK and UKCCCR Guidelines (1998) were followed throughout.

### Tumour system

MAC 15A, a rapidly growing poorly differentiated adenocarcinoma of the colon was used in this study (Phillips et al, 1988). MAC 15A cells were routinely maintained in tissue culture. Prior to chemotherapy experiments, $1 \times 10^6$ cells in sterile saline were injected subcutaneously into the flank of each mouse (Laws et al, 1995).

### Chemotherapy

Chemotherapy began when the tumours had reached a size that could be reliably measured i.e. 3–4 days after implantation. Test compounds were administered by a single intraperitoneal injection to groups of 10 mice and the effects of therapy were assessed by daily 2-dimensional caliper measurements of the tumours. Tumour volumes were calculated from the formula $a^2 \times b/2$, where a is the smaller and b is the larger diameter of the tumour. Tumour volumes were normalized with respect to their initial volumes, graphs of the relative tumour volume against time were plotted and Mann-Whitney U tests performed to determine the statistical significance of any differences in growth rate (tumour-volume doubling time) between control and treated groups.

### Histology

At the end of the experiments the animals were killed and histological examination of the tumours was carried out. In addition, two animals per treatment group were killed 24 hours after treatment and their tumours were examined histologically. Drug induced haemorrhagic necrosis was quantified using an image analysis system (Seescan, Cambridge, UK).

### Vascular assessment

In order to assess effects of treatment with vinflunine on functional vasculature 5 groups of 12 tumour-bearing mice were set up. One group was used as a control and the other 4 were treated with different doses of vinflunine ranging from 10 to 50 mg $kg^{-1}$. To assess the vascular effects of vinorelbine two groups of tumour-bearing mice were set up; one group as a control and the other treated with 10 mg $kg^{-1}$ vinorelbine. Treatment was initiated when the tumours had reached a similar size to those used in the chemotherapy experiments described above i.e. had an established blood vasculature. Hoechst 33342 (bisBenzimide) was used to assess functional tumour vasculature (Quinn et al, 1992; Cowen et al, 1995). This compound was dissolved in sterile saline and injected intravenously via the tail vein at a dose of 40 mg $kg^{-1}$, 2, 4, 6 and 24 hours after vinflunine treatment or 2 hours after vinorelbine treatment. One minute after injection of the vascular marker the mice were killed by cervical dislocation (3 mice at each time point), dissected and the tumour resected. Tumours were wrapped in aluminium foil and immediately immersed in liquid nitrogen and stored at −80˚C until sectioning.

Frozen sections (7 µm) were cut on a cryostat (Bright Instrument Co Ltd, Huntingdon, UK) and air-dried. 5 random sections were obtained from each tumour and they were examined under UV illumination using a Vickers microscope at a magnification of 250. The fluorescent dye concentrates in the nuclei of the endothelial cells, so functional vasculature may be observed as fluorescence and the area of fluorescence was determined by counting the number of positive squares using a 400 square graticule from 5 random fields in each section. Comparisons were made between percentage vasculature in control and treated tumours.

### HUVEC assays

Primary endothelial cells derived from the human umbilical vein (HUVECs, a gift from Dr A Graham, University of Bradford) were cultured in Medium 199 supplemented with 10% human serum, 1 mM sodium pyruvate, 50 Iu $ml^{-1}$ penicillin, 50 g $ml^{-1}$ streptomycin and 2 mM L-glutamine. HUVECs were grown in gelatine coated flasks. Briefly, 5 ml gelatin was added to each tissue culture flask and was allowed to coat the bottom. The flasks were then placed in an incubator for 30 minutes after which time excess gelatin was removed. Hanks' balanced salts solution (HBSS) was used to wash the flasks which were then ready for use.

In order to assess the influence of vinflunine on apoptosis, HUVECs were grown in gelatin coated 6-well plates and exposed to 3 concentrations around the $IC_{50}$ for tumour cells (Kruczynski et al, 1998b) for 4, 8 or 24 hours. Following incubation with the drug, media were removed and kept and adherent cells were detached from the wells with trypsin. These cells were added to the previously collected media. Cells were pelleted by centrifugation and resuspended in 50 µl HBSS. Cells were smeared on APES-coated slides, allowed to dry and fixed in 95% ethanol for 20 minutes. Cell smears were placed in distilled water before staining in a 1% solution of Hoechst 33342 for 20 minutes in the dark at room temperature. After staining, slides were washed in distilled water (2 minutes) and mounted with an aqueous mountant, glycerol/PBS (Citifluor, Agar Scientific). Apoptotic cells were detected by dark field, UV microscopy under oil (×1000).

### RESULTS

### Tumour growth studies

The anti-tumour effects of test compounds against the MAC 15A subcutaneous tumour are presented in Figure 1A. Each treatment resulted in a statistically significant tumour growth delay and although, from the figure, it appears that the highest dose of vinflunine (60 mg $kg^{-1}$) tested was more effective than the other treatments, there was toxicity in this group. In this group, 2 out of 10 mice were killed for humane reasons and there was an additional drug-associated death. There were also two drug-associated deaths in the vinorelbine treated (10 mg $kg^{-1}$) group. A second experiment examined slightly lower doses of vinorelbine and vinflunine (8 and 50 mg $kg^{-1}$ respectively) in an attempt to optimize the single dose level and the results are presented as Figure 1B. Both these treatments produced significant anti-tumour effects and vinflunine was well tolerated with no obvious signs of toxicity at 50 mg $kg^{-1}$. However, vinorelbine treatment at this dose of 8 mg $kg^{-1}$ still resulted in definite body weight loss and obvious diarrhoea, although body weights were recovering by the end of the experiments.

### Morphological effects

Examination of histological sections of the tumours suggested that treatment with either vincristine, vinorelbine or vinflunine all caused an increase in the percentage of necrosis compared to controls. In the untreated tumours most of the sections were of normal healthy appearance (Figure 2). An example of the morphological appearance of the tumours from vincristine-treated (1.5 mg $kg^{-1}$) tumours is shown in Figure 3 and although necrosis was present, viable vasculature was easily definable. Haemorrhagic necrosis was particularly obvious in the group of mice treated with vinorelbine (10 mg $kg^{-1}$) (Figure 4A) and was extensive in the groups treated with vinflunine (50 mg $kg^{-1}$) (Figure 4B). There was clear evidence of vascular damage in tumours taken from mice treated with either vinorelbine (8 mg $kg^{-1}$) or vinflunine (50 mg $kg^{-1}$), as shown in Figure 5.

*© 2001 Cancer Research Campaign* 

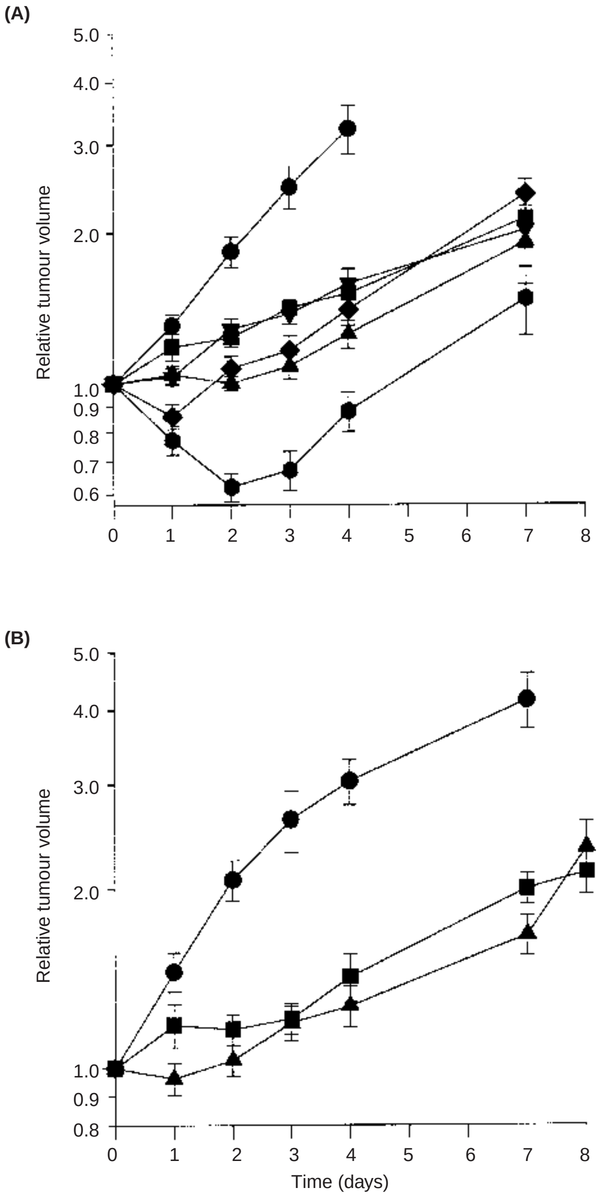

**Figure 1** (**A**) Anti-tumour effects of vinflunine, vinorelbine and vincristine in the MAC 15A subcutaneous tumour model; untreated controls (●), 1.5 mg $kg^{-1}$ vincristine (■), 10 mg $kg^{-1}$ vinorelbine (▲), 20 mg $kg^{-1}$ vinflunine (▼), 40 mg $kg^{-1}$ vinflunine (◆) and 60 mg $kg^{-1}$ vinflunine (●). (**B**) Optimized single dose concentrations of vinorelbine and vinflunine; untreated controls (●), 8 mg $kg^{-1}$ vinorelbine (■) and 50 mg $kg^{-1}$ vinflunine (▲). Points represent mean ±SEM

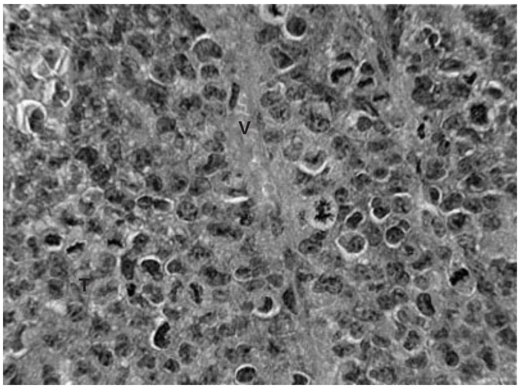

**Figure 2** Morphological appearance of an untreated MAC 15A subcutaneous tumour. T represents viable tumour, V indicates a blood vessel

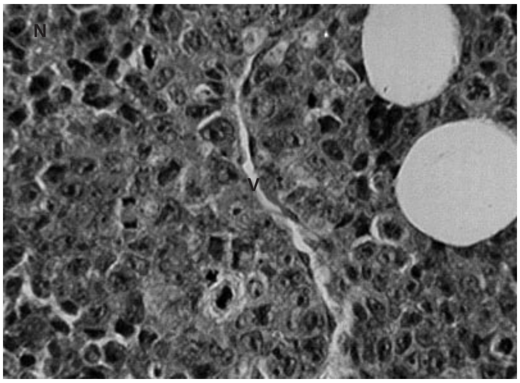

**Figure 3** Morphological appearance of a MAC 15A tumour 24 hours after treatment with 1.5 mg $kg^{-1}$ vincristine. N indicates an area of necrosis, V indicates a blood vessel

Control MAC 15A tumours displayed 34.9% (SD 11.86) haemorrhagic necrosis. Tumours treated with 10 mg $kg^{-1}$ vinorelbine showed a significantly increased percentage of necrosis at 71.0% (SD 5.2). Treatment with 50 mg $kg^{-1}$ vinflunine also resulted in a significantly increased percentage of necrosis compared to the controls and this value was marginally higher than that determined for vinorelbine at 73.5% (SD 15.6).

### Vascular shutdown studies

The fluorescent dye perfusion studies carried out confirmed our earlier observation (Quinn et al, 1992; Cowen et al, 1995) that control MAC 15A tumours were highly vascular. Analysis of frozen sections of tumours from mice treated with vinflunine at 50 mg $kg^{-1}$ indicated a very clear vascular shutdown within the first 4 hours following treatment. This vascular shutdown remained over the 24-hour period evaluated (Figure 6A). No fluorescence was seen within the body of the tumours treated with this dose of vinflunine (50 mg $kg^{-1}$) and fluorescence was limited to the tumour periphery. In the next series of experiments functional vasculature was evaluated following administration of the 3 lower doses of vinflunine ranging from 10 to 40 mg $kg^{-1}$. Analysis of frozen sections of control tumours for fluorescence indicated a mean count for functional vascularity of 16.01% (Figure 6B). Comparison with tumours taken from vinflunine-treated groups of mice demonstrated vascular effects at each dose level employed, although anti-vascular effects appeared more rapid with increased dosage. A mean of only 1.03% (SD 3.86) functional vasculature remained two hours after treatment with 10 mg $kg^{-1}$ vinorelbine.

Overall these data indicate that vinflunine caused vascular shutdown in the transplantable murine adenocarcinoma model MAC 15A and these effects are obtained at doses below the optimum effective dose.

### HUVEC assays

4 and 8 hours after exposure to 0.05, 0.5 or 1 μM vinflunine, HUVECs were not undergoing apoptosis although even at the

© 2001 Cancer Research Campaign

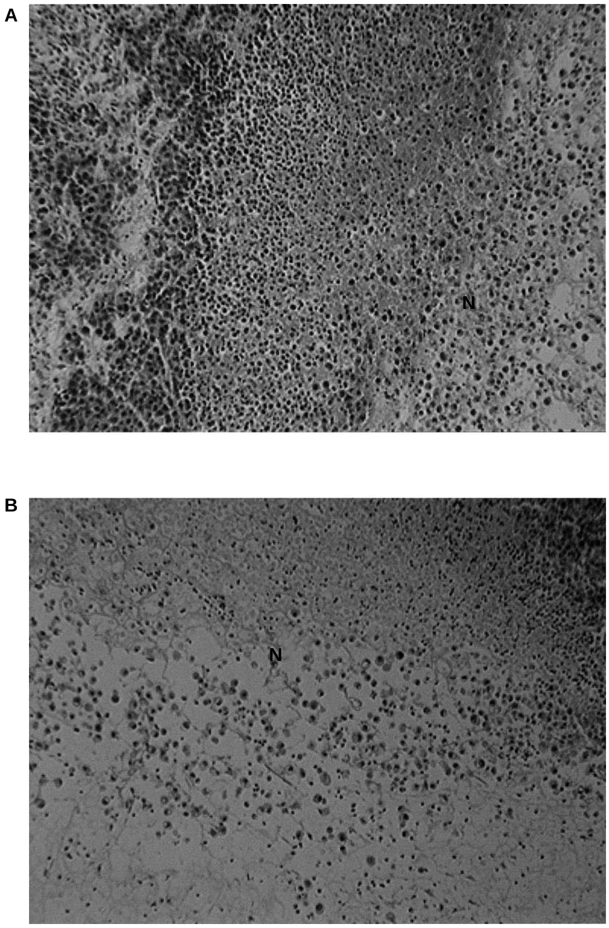

**Figure 4** (**A**) Morphological appearance of a MAC 15A tumour 24 hours after treatment with 10 mg $kg^{-1}$ vinorelbine. (**B**) Morphological appearance of a MAC 15A tumour 24 hours after treatment with 50 mg $kg^{-1}$ vinflunine. N indicates an area of necrosis

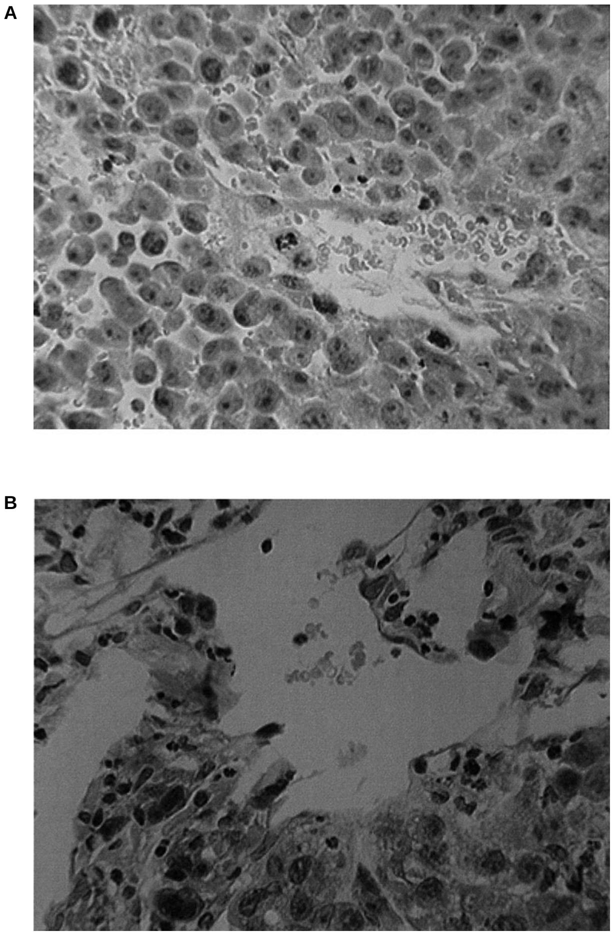

**Figure 5** Vascular damage in tumours taken from mice treated with (**A**) 8 mg $kg^{-1}$ vinorelbine and (**B**) 50 mg $kg^{-1}$ vinflunine lowest concentration, cell shape changes were evident. There was clear evidence of apoptosis after 24 hours but only after exposure to the highest (1 μM) concentration of vinflunine (Figure 7).

### DISCUSSION

The *Vinca* alkaloid family is one of the principal groups of antitumour compounds used routinely in the clinic today (Budman, 1992, 1997). Previously, members of this family such as vincristine and vinblastine have been shown to mediate their antitumour activities via an antivascular mechanism (Hill et al, 1993). Novel derivatives of these compounds, namely vinorelbine and vinflunine, have been shown to display significant antitumour activity (Johnson et al, 1996; Kruczynski et al, 1998a; Hill et al, 1999). Indeed, vinorelbine is a clinically active compound and is currently widely used in the treatment of cancer, especially in non-small cell lung cancer and breast cancer (Berthaud et al, 1992; Fumoleau et al, 1993; Le Chevalier et al, 1994; Romero et al, 1994; Fumoleau et al, 1995; Budman, 1997). Vinflunine is currently in Phase I trials in Europe. Results presented here demonstrate that both these new *Vincas* also mediate their antitumour activities, at least in part, via an antivascular mechanism.

In summary, the results indicate that vinflunine exerts antitumour effects against the MAC 15A transplantable murine adenocarcinoma model and is at least as effective as vincristine and vinorelbine using single-dose schedules. Morphological changes, particularly the appearance of haemorrhagic necrosis, accompanying tumour growth delay suggested a possible anti-vascular effect and this was confirmed by the Hoechst 33342 perfusion study that showed vascular shutdown over a minimum of 24 hours. Vascular shutdown in the MAC 15A tumours was obtained even at doses below the optimum effective single dose. The potential importance of inducing vascular shutdown within tumours at doses less than the maximum tolerated dose (MTD) has been emphasized in studies of combretastatin A-4, which has been shown recently to have a wide therapeutic window in several in vivo experimental cancer models, including the MAC 15A murine tumour tested here (Dark et al, 1997; Grosios et al, 1999; Tozer et al, 1999). In addition our results indicate that vinflunine appears to demonstrate both enhanced antitumour activity and vascular effects relative to vinorelbine. Combretastatin A-4 has been demonstrated to induce endothelial cell shape changes in vitro (Grosios et al, 1999) and similar changes in HUVEC shape were observed in this study. However endothelial cell apoptosis although clear at 24 hours did not occur following exposure to vinflunine for up to 8 hours. Endothelial cell shape change rather than apoptosis may be the primary event resulting in the rapid vascular shutdown seen in tumours in vivo after treatment with vinflunine.

*© 2001 Cancer Research Campaign*

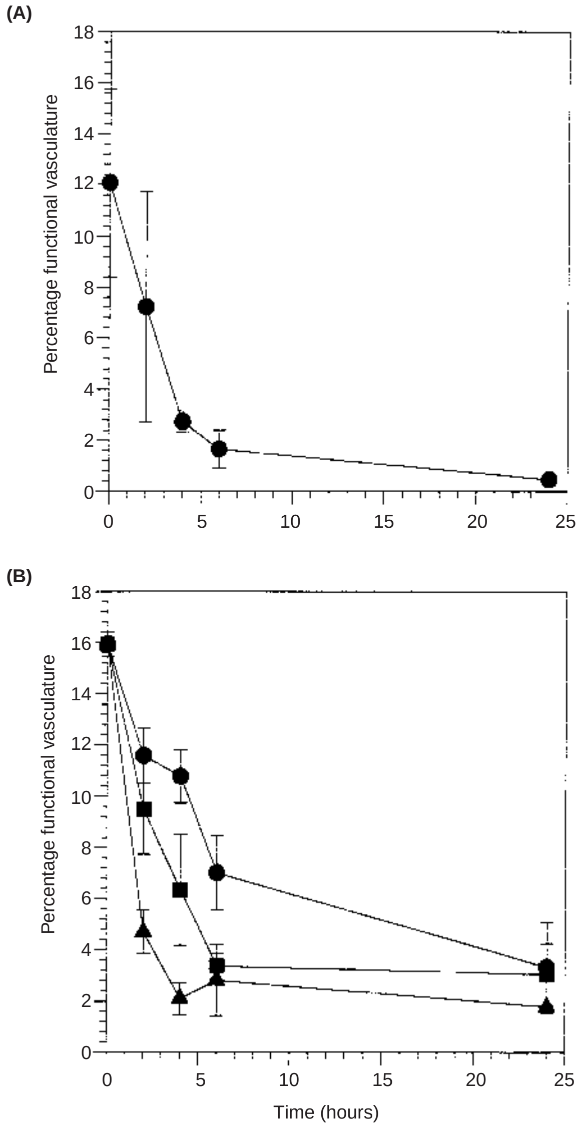

**Figure 6** Hoechst 33342 vascular perfusion studies using 50 mg kg$^{-1}$ vinflunine (●) administered via a single intraperitoneal injection. *n*=3, (**B**) using 10 mg kg$^{-1}$ (●), 20 mg kg$^{-1}$ (■) and 40 mg kg-1 (▲). Points represent mean SEM

These data may have definite implications for combination chemotherapy with vinflunine, since it may be possible to exploit tumour blood flow changes so as to improve drug exposure characteristics or to alter tumour microenvironmental properties for prodrug activation. With regard to the development of an active chemotherapy regimen, it would be advantageous to investigate these potential anti-vascular effects in the current phase I/II clinical trials, particularly since it has been demonstrated that vascular effects are seen with vinflunine below the MTD. In fact earlier scheduling experiments in mice bearing P388 leukaemia have shown that doses of vinflunine up to 40 mg kg$^{-1}$ per injection are effective in 2 weekly or 4 weekly schedules up to a total dose of 240 mg kg$^{-1}$ (Kruczynski et al 1998a,b). Although the most effective schedule was 4 weekly doses of 40 mg kg$^{-1}$ totalling 160 mg kg$^{-1}$. The present investigation has indicated tumour vascular effects well below these doses. Combination of vinflunine with other drugs with different mechanisms of action at doses below their MTDs may thus result in a synergy of action against the tumour with minimal host toxicity.

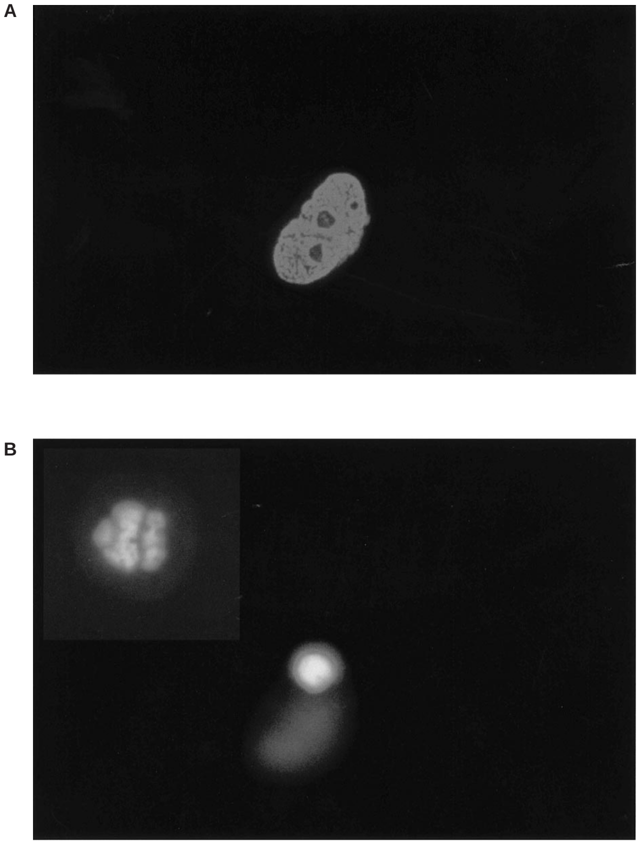

**Figure 7** Appearance of apoptosis in HUVECs stained with Hoechst 33342 **(A)** control nucleus **(B)** exposure to 1 μM vinflunine for 24 hours demonstrating condensed chromatin; insert indicates apoptotic bodies

### ACKNOWLEDGEMENTS

We are grateful to War on Cancer and the Institut de Recherche Pierre Fabre for their support.

### REFERENCES

Algire GH, Legallais FY and Anderson BF (1954) Vascular reactions of normal and malignant tissues in vivo. VI. The role of hypotension in the action of components of podophyllin on transplanted sarcomas. *J Natl Cancer Inst* **14**: 879–887

Baguley BC, Holdaway KH, Thomsen LL, Zhuang L and Zwi LJ (1991) Inhibition of growth of colon 38 adenocarcinoma by vinblastine and colchicine. Evidence for a vascular mechanism. *Eur J Cancer* **27**: 482–487

Berthaud P, Le Chevalier T, Ruffie P, Baldeyrou P, Arriagada R, Besson F and Tursz T (1992) Phase I–II study of vinorelbine (Navelbine) plus cisplatin in advanced non-small cell lung cancer. *Eur J Cancer* **28**: 1863–1865

Budman DR (1992) New vinca alkaloids and related compounds. *Semin Oncol* **19**: 639–645

Budman DR (1997) Vinorelbine (Navelbine): a third-generation Vinca alkaloid. *Cancer Invest* **15**: 475–490

Chaplin DJ and Dougherty GJ (1999) Tumour vasculature as a target to cancer therapy. *Br J Cancer* **80**(1): 57–64

Cowen SE, Bibby MC and Double JA (1995) Characterisation of the vasculature within a murine adenocarcinoma growing in different sites to evaluate the potential of vascular therapies. *Acta Oncol* **34**: 357–60

Dark GG, Hill SA, Prise VE, Tozer GM, Pettit GR and Chaplin DJ (1997) Combretastatin A-4, an agent that displays potent and selective toxicity toward tumour vasculature. *Cancer Res* **57**: 1829–1834

Fahy J, Duflos A, Ribet JP, Jacquesy JC, Berrier C, Jouannetaud MP and Zunino F (1997) *Vinca* alkaloids in superacid media: a method for creating a new family of antitumor derivatives. *J Am Chem Soc* **119**: 8576–8577

*© 2001 Cancer Research Campaign*

Fumoleau P, Delgado FM, Delozier T, Monnier A, Gil Delgado MA, Kerbrat P, Garcia-Giralt, Keiking R, Namer M, Closon MT, Goudier MJ, Chollet P, Lecourt L and Montcuquet P (1993) Phase II trial of weekly intravenous vinorelbine in first-line advanced breast cancer chemotherapy. *J Clin Oncol* **11**: 1245–1252

Fumoleau P, Delozier T, Extra JM, Canobbio L, Delgado FM and Hurteloup P (1995) Vinorelbine (Navelbine) in the treatment of breast cancer: the European experience. Semin Oncol 22 [2 suppl 5]: 22–29

Grosios K, Holwell SE, McGown AT, Pettit GR and Bibby MC (1999) in vivo and in vitro evaluation of combretastatin A-4 and its sodium phosphate prodrug. *Br J Cancer* **81**: 1318–1327

Hayes AJ, Li LY and Lippman ME (1999) Antivascular therapy: a new approach to cancer treatment. *BMJ* **318**: 853–856

Hill SA, Longergan SJ, Denekamp J and Chaplin DJ (1993) *Vinca* alkaloids: anti-vascular effects in a murine tumour. *Eur J Cancer* **29**: 1320–1324

Hill BT, Fiebig HH, Waud WR, Poupon MF, Colpaert F and Kruczynski A (1999) Superior *in vivo* experimental antitumour activity of vinflunine, relative to vinorelbine, in a panel of human tumour xenografts. *Eur J Cancer* **35**: 512–520

Johnson SA, Harper P, Hortobagyi GN and Pouillart P (1996) Vinorelbine: an overview. *Cancer Treat Rev* **22**: 127–142

Kruczynski A, Colpaert F, Tarayre JP, Mouillard P, Fahy J and Hill BT (1998a) Preclinical *in vivo* antitumour activity of vinflunine, a novel fluorinated *Vinca* alkaloid. *Cancer Chemother Pharmacol* **41**: 437–447

Kruczynski A, Barret J-M, Etievant C, Colpaert F, Fahy J and Hill BT (1998b) Anti-mitotic and tubulin-interactive properties of vinflunine, a novel fluorinated vinca alkaloid. *Biochem Pharmacol* **55**: 635–648

Laws AL, Matthew AM, Double JA and Bibby MC (1995). Preclinical *in vitro* and *in vivo* activity of 5,6-dimethylxanthenone-4-acetic acid. *Br J Cancer* **71**: 1204–1209

Le Chevalier T, Brisgand D, Douillard JY, Pujol JL, Alberola V, Monnier A, Riviere A, Lianes P, Chomy P, Cigolari S, Gottfried M, Ruffie P, Panizo A, Gaspard MH, Ravaioli A, Besenval M, Besson F, Martinez A, Berthaud P and Tursz T (1994) Randomized study of vinorelbine and cisplatin versus vindesine and cisplatin versus vinorelbine alone in advanced non-small-cell lung cancer: Results of a European multicenter trial including 612 patients. *J Clin Oncol* **12**: 360–367

Ludford RJ (1948) Factors determining the action of colchicine on tumour growth. *Br J Cancer* **2**: 75–86

Phillips RM, Bibby MC and Double JA (1988) Experimental correlations of *in vitro* drug sensitivity with *in vivo* responses to Thio TEPA in a panel of murine: tumours. *Cancer Chemother Pharmacol* **21**: 168–172

Quinn PK, Bibby MC, Cox JA and Crawford SM (1992) The influence of hydralazine on the vasculature, blood perfusion and chemosensitivity of MAC tumours. *Br J Cancer* **66**: 323–330

Romero A, Rabinovich MG and Vallejo CT (1994) Vinorelbine as first-line chemotherapy for metastatic breast cancer. *J Clin Oncol* **12**: 336–341

Tozer GM, Prise VE, Wilson J, Locke RJ, Vojnovic B, Stratford MRL, Dennis MF and Chaplin DJ (1999) Combretastatin A-4 phosphate as a tumor vascular-targeting agent: early effects in tumors and normal tissues. *Cancer Res* **59**: 1626–1634

UKCCCR Guidelines for the Welfare of Animals in Experimental Neoplasia (1998). *Br J Cancer* **77**: 1–10

© 2001 Cancer Research Campaign 
